# Therapeutic potential of naringin in improving the survival rate of skin flap: A review

**DOI:** 10.3389/fphar.2023.1128147

**Published:** 2023-03-02

**Authors:** Yincang Wang, Xiaodong Li, Hang Lv, Lin Sun, Bo Liu, Xiaofeng Zhang, Xilin Xu

**Affiliations:** ^1^ Heilongjiang University of Chinese Medicine, Harbin, China; ^2^ The Third Department of Orthopedics and Traumatology, The Second Affiliated Hospital of Heilongjiang University of Chinese Medicine, Harbin, China; ^3^ Teaching and Research Section of Orthopedics and Traumatology, Heilongjiang University of Chinese Medicine, Harbin, China

**Keywords:** naringin, skin flap survival, angiogenesis, inflammation, VEGF

## Abstract

Naringin is the main component of Drynaria. Modern pharmacological studies have shown that naringin has a wide range of pharmacological activities, including antioxidant, anti-inflammatory, anti-apoptotic, anti-ulcer, and anti-osteoporosis effects. Its therapeutic effects have been observed in various clinical models, such as atherosclerosis, cardiovascular diseases, diabetes, neurodegenerative diseases, and rheumatic diseases. This review investigates the pharmacological effects of naringin and the associated mechanisms in improving flap survival. This review will also provide a reference for future rational application of naringin, especially in research to improve flap survival.

## 1 Introduction

Flap grafting is widely used for the repair and reconstruction of clinical tissue defects (Bordianu et al., 2018; Adani et al., 2022; Hart et al., 2022) and is one of the important surgical techniques for skin defect repair. Among them, random flap grafting is commonly used to repair refractory wounds, accidental trauma injuries, cancer resection, and skin defects caused by diabetes mellitus. The random flap, also called the arbitrary flap, does not contain arterial axial vessels but only the dermal vascular network, subdermal vascular network, and sometimes the subdermal vascular network. Therefore, one must pay attention to the limitation of the length-to-width ratio during flap grafting so that the range of cut flaps is limited by a certain length-to-width ratio, with the general ratio of length to the width being 1:1 to 1.5:1 (Saint-Cyr et al., 2009; Memarzadeh et al., 2016). Although the design and surgical techniques of flaps are constantly improving, cases of partial or total necrosis, a common complication after repair, often occur in clinical practice. Partial or total necrosis may eventually result in disability of the affected limb and cause great physical and psychological pain to the patient, thus limiting its application. Therefore, it is important to ensure a high survival rate of the flap to expand clinical applications and improve clinical outcomes (van den Heuvel et al., 2009). A key factor in flap survival is good circulation (Brumberg et al., 2021). Blood supply to the flap is generally through the vascular ganglion within the tip bed of the flap and neovascularization from the tip to the distal end; however, the flap lacks adequate nutrient supply because of the lack of axial vessels and relies on transcutaneous vessels emanating from the tissue tip for blood supply. Therefore, flap necrosis is highly probable after distal displacement of some flaps due to malnutrition (Deng et al., 2019). Flap survival is also influenced by metabolic factors and the degree of tolerance of the tissue to ischemia and hypoxia (Fan et al., 2021; Huang et al., 2021). Changes in toxic products such as lipid peroxides and reactive oxygen species (ROS) in tissues and blood vessels caused by ischemia-reperfusion injury after flap repair can also lead to flap necrosis (Carroll and Esclamado, 2000; van den Heuvel et al., 2009), so it is important to improve the flap microcirculation, increase angiogenesis, and reduce the damage caused by flap ischemia-reperfusion to improve flap survival.

Free flap grafting has a success rate of 90%–95% (Qiu et al., 2019), whereas the postoperative necrosis rate of tipped flaps is 20%–33% (Moran and Serletti, 2001), which causes great harm to patients. It may prolong hospitalization, increase the number of surgeries and treatment costs, directly affect the therapeutic outcome of flap surgery, and increases the financial burden on the patient. There is still no treatment method to completely solve the problem of necrosis after flap repair. However, in recent years, phytochemicals have been found to play an important role in the treatment of flap necrosis by promoting the formation of new blood vessels, preventing flap necrosis, and accelerating healing after flap repair, thus providing new ideas significant for the treatment of flap necrosis (Weinzierl et al., 2022). In one study, Muscone affected random flap survival in rats. In that study, compared to the control group, the experimental group showed an increase in flap survival area, flap angiogenesis, vascular endothelial growth factor (VEGF) expression, and superoxide dismutase (SOD) activity, while malondialdehyde (MDA) content was reduced in the flaps treated in the experimental group in addition to lowered oxidative stress and the rate of apoptotic cell death. These results indicated that it could improve flap survival (Kailiang et al., 2016). In another study, flap survival could be improved by Huangqi injection; compared to the control group, the mean survival area of flaps in the experimental group was found to be significantly higher, along with a higher expression of VEGF, SOD, the development of microvessels, and decreased level of MDA (Cai et al., 2015). Recently, He et al. (2020) showed that azadirachtin A inhibited the chemotaxis and adhesion of neutrophils, reduced the levels of inflammatory factors tumor necrosis factor-α (TNF-α), interleukin-6(IL-6), and interleukin-1β(IL-1β), increased flap survival area, improved flap blood supply, and reduced ischemia-reperfusion injury by inhibiting the nuclear factor kappa-B (NF-κB) signaling pathway and upregulating the expression of SOD and VEGF to improve tissue blood supply and metabolism to improve flap survival (He et al., 2020). Another study showed that andrographolide positively affects randomized flap survival through the phosphatidylinositol-3-kinase/protein kinase B (PI3K/Akt) signaling pathway (Jiang et al., 2021). A study by Li et al. (2022) found that formononetin activates nuclear translocation of nuclear factor-E2-related factor 2(Nrf2) and nuclear translocation-mediated antioxidant effects, inhibits Akt expression, increases SOD and oxidase activity, enhances VEGF expression, reduces IL-1β and TNF-α levels, reduces oxidative stress and inflammation, improves flap microcirculation, and promotes flap angiogenesis, thereby improving random flap survival. These studies show that phytochemicals have significant advantages in promoting the formation of new blood vessels, treating flap necrosis, and healing after flap repair, proving the great potential of phytochemicals in treating flap necrosis and having important implications for future research on flap necrosis treatment.

**FIGURE 1:**
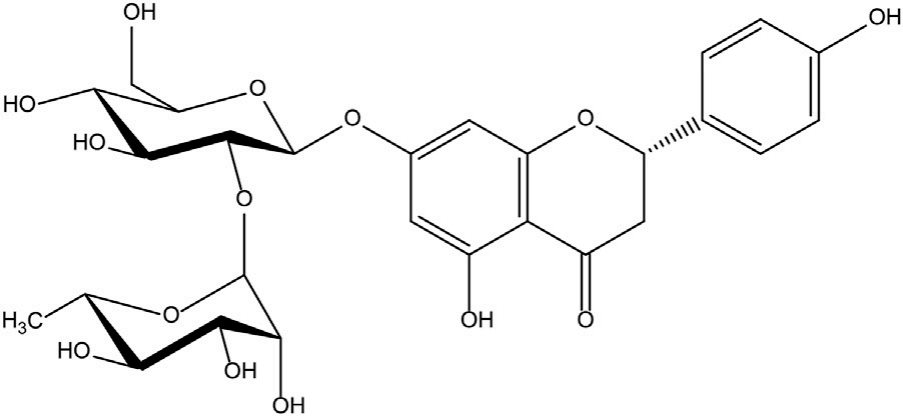
The chemical structure of naringin.

**FIGURE 2:**
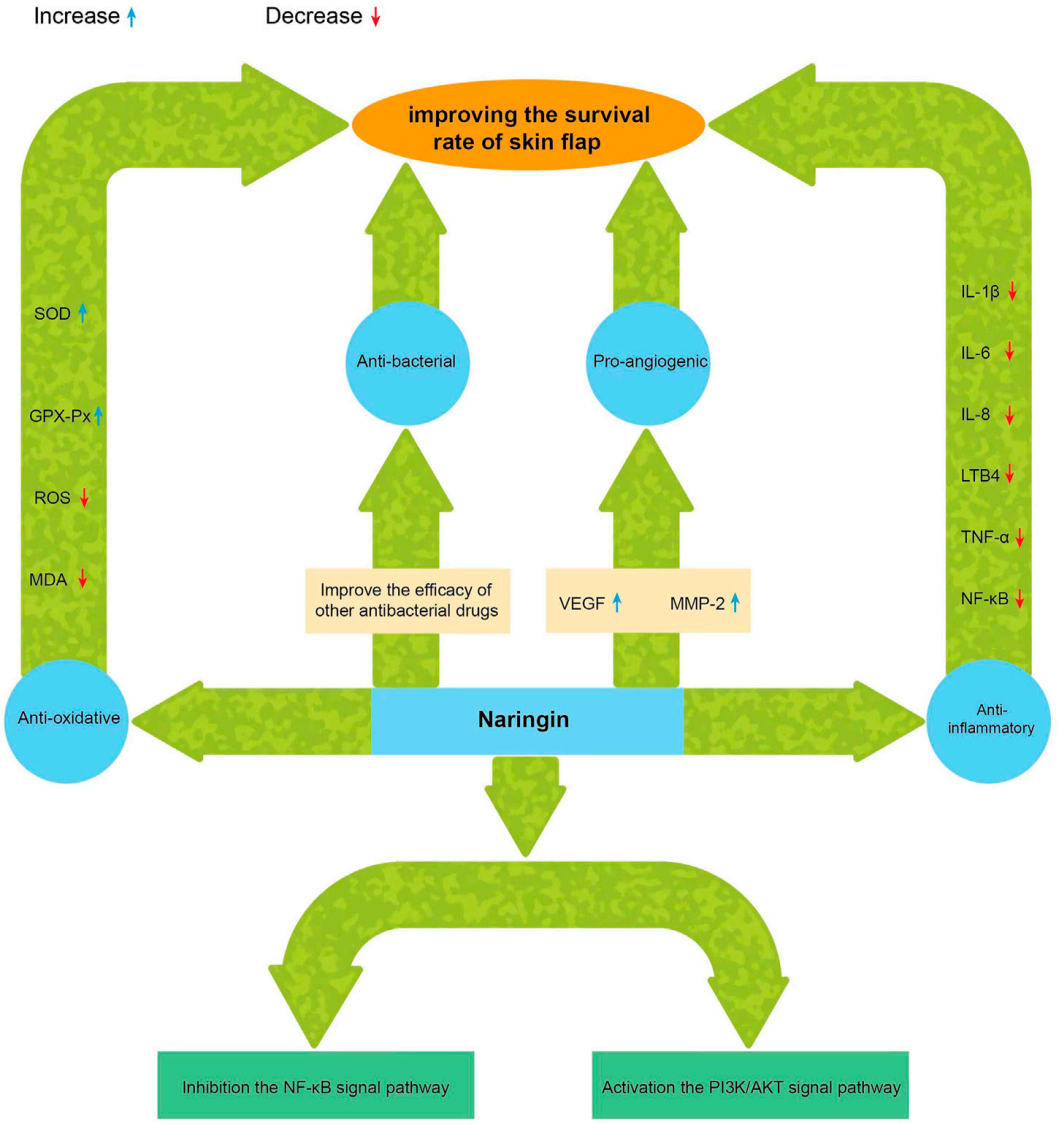
Beneficial effect of naringin on improving the survival of skin flap.

The chemical name of naringin, a flavonoid, is 4′, 5,7-trihydroxy flavone 7-rhamnoside (the molecular formula is C_27_H_32_O_14_ and a molecular weight of 580.4 g/mol) (Figure 1); it contains the basic structure of flavonoids, and two rhamnose units are attached to its glycosidic moiety (Alam et al., 2014). Naringin is obtained from citrus fruits (Tripoli et al., 2007) and is one of the main active components of citrus herbs (Alam et al., 2014; Zhang et al., 2014)and Drynaria fortunei (Guo et al., 2011; Chen et al., 2021). Modern pharmacological research found that naringin has antioxidant (Singh et al., 2020), antibacterial (Adamczak et al., 2019), anti-inflammatory (Mohanty et al., 2020),anti-osteoporosis (An et al., 2016), anti-tumor (Ghanbari-Movahed et al., 2021), and improves myocardial damage (Sun et al., 2019), liver damage (Rodríguez et al., 2018), and blood lipids (Raja Kumar et al., 2019), and prevents diabetes and obesity (Shen et al., 2012; Alam et al., 2014). Single doses of naringin and naringenin gave by intravenous push and oral routes to rats and were present in the blood mainly as naringin glucuronide and sulfate conjugates (Fang et al., 2006). Naringin circulates in the hepatic-intestinal system and is excreted *via* the liver or bile (Ribeiro, 2011; Tsai and Tsai, 2012). Naringin alone, or in combination with other drugs, may be useful in the treatment of ischemic necrosis of flaps and has great potential in improving flap viability due to its single component; It is easily available, has a long history of use, diverse pharmacological effects, and is non-toxic (Budel et al., 2020; Li et al., 2020). There are few clinical studies of phytochemicals in flap surgery, and naringin, with its pleiotropic and tissue-protective effects, could be ideal for improving flap survival (Figure 2). Although naringin has numerous pharmacological effects, the research related to the role of naringin in improving flap survival is at the initial stage, and the associated mechanism is still being explored by researchers worldwide. Therefore, this review summarizes the up-to-date information on the pharmacological effects of naringin in improving flap survival and its mechanism, with a view to providing a reference for further research and hope to profoundly impact the clinical treatment of flap necrosis in the future.

IL-1β = interleukin-1β; IL-6 = interleukin-6; IL-8 = interleukin-8; TNF-α = tumor necrosis factor-α; LTB4 = leukotriene B4; NF-κB =nuclear factor kappa-light-chain-enhancer of activated B cells; VEGF = vascular endothelial growth factor; MMP-2 = matrix metalloproteinase-2; SOD =superoxide dismutase; GSH-Px = glutathione peroxidase MDA = malondialdehyde; ROS = reactive oxygen species.

## 2 Naringin promotes angiogenesis and increases the flap survival rate

Neovascularization is closely related to flap survival. Angiogenesis, the development of new microvessels from existing microvessels, is regulated by a balanced interaction of pro- and anti-angiogenic factors and requires close interaction between endothelial cells and perivascular cells (Chiaverina et al., 2019). VEGF is a pro-angiogenic factor that acts specifically on vascular endothelial cells, promoting vascular endothelial cell proliferation, increasing vascular permeability, reducing ischemia-reperfusion injury in flaps, and promoting neoangiogenesis (Ferrara, 2001). The study showed that exogenous VEGF could effectively improve blood circulation in rat random flap graft tissues, thereby increasing flap survival (Vourtsis et al., 2012). Later, Fang et al. (2014) suggested that VEGF improves casual flap survival locally by inducing angiogenesis and promoting distal flap revascularization. Acute and sustained VEGF on flap and skin graft survival has been shown to increase neovascularization and improve early flap survival (Zhang and Lineaweaver, 2011). VEGF also protected flaps from ischemia-reperfusion injury by modulating pro-inflammatory cytokines and inhibiting cytotoxic nitric oxide production, thereby improving flap survival in rats (Pang et al., 2003). Naringin has the ability to enhance VEGF expression and promote neoangiogenesis. Several major components of Drynaria, including naringenin, increase matrix metallopeptidase-2 (MMP-2) activity *in vitro* and *in vivo* by regulating the balance of MMP-2 and tissue inhibitors of MMP-2, activating VEGF and its receptor (VEGFR) expression, and thus promoting angiogenesis and cell migration (Huang et al., 2018). When assessing the effect of naringin in improving the survival rate of random flaps in rats, flap angiography and laser Doppler imaging revealed that the mean vascular density and the number of vessels were significantly increased in the naringin group compared with the control group, indicating that naringin could indeed increase the number of vessels in damaged tissues by enhancing VEGF expression, thus improving flap survival (Cheng et al., 2017). Naringin significantly increased the expression of VEGF and VEGFR⁃2 in fracture scabs of devitalized rats and promoted early angiogenesis of fracture healing in devitalized rats (Song et al., 2017). In another study, naringin was able to activate the PI3K/Akt signaling pathway through the CXC motif chemokine ligand 12/CXC motif chemokine receptor 4 axis to mediate enhanced endothelial progenitor cell proliferation and tube formation, demonstrating the potential of naringin as a novel drug to treat ischemic diseases (Zhao et al., 2018).

In summary, several studies have demonstrated that naringin can promote neoangiogenesis. The mechanism may be related to the ability of naringin to promote neoangiogenesis through the PI3K/Akt signaling pathway and enhanced VEGF expression; non-etheless, the specific signaling pathways have not been precisely investigated yet and need to be explored.

## 3 Naringin inhibits the inflammatory response generated during ischemia-reperfusion of the flap

Reperfusion is essential for flap survival. During free flap grafting, the tissue needs to endure a period of complete ischemia. After the flap is grafted from the donor to the recipient site to complete the vascular anastomosis, when blood perfusion is re-established, the generation of ROS and inflammatory responses can lead to additional cellular damage (Siemionow and Arslan, 2004). The damage during ischemia-reperfusion of the flap is mainly caused by two mechanisms. First, direct damage to the vascular endothelium by superoxide radicals, leading to lipid peroxidation, destruction of membrane proteins, and increased cell permeability, ultimately leading to cytoplasmic swelling and dysfunction. In an ischemic state, oxygen radicals accumulate in large quantities in the tissue, aggravating the inflammatory response and further causing damage to the flap tissue after ischemia-reperfusion (Wu et al., 2018). Naringin could scavenge free radicals and increase SOD and glutathione peroxidase activity *in vivo* by regulating the expression of antioxidant defense proteins (Hager-Theodorides et al., 2021). Naringin exerts its total antioxidant capacity in the body through its activity (El-Desoky et al., 2018; Wang et al., 2020). Naringin improved coronary ischemia-reperfusion injury in rats by scavenging free radicals and antioxidant properties (Liu et al., 2022), and it was also protective against ischemia-reperfusion-induced oxidative stress in rat testes (Akondi et al., 2011). Another study revealed the protective effect of naringin against myocardial ischemia-reperfusion injury in rats (Li et al., 2021). These studies demonstrated the free radical scavenging and antioxidant effects of naringin, which can significantly improve intracellular antioxidant capacity, reduce oxidation levels, and effectively reduce the damage caused by ischemia-reperfusion. Second, a large accumulation of neutrophils during ischemia-reperfusion damages the tissue and the cell membrane. Blood in ischemia-reperfused tissues increases metabolites of arachidonic acid; when it is restored due to the degradation of membrane phospholipids, and the vascular endothelium adheres to a large number of leukocytes, damaging the endothelial cells more seriously (Siemionow and Arslan, 2004), and the produced substances further aggravate the local inflammatory response. Naringin possesses good inflammatory inhibitory activity and inhibits cyclooxygenases and lipoxygenase oxidases involved in the biosynthesis of arachidonic acid-derived mediators (Manthey et al., 2001). In an inflammation model of guinea pig with cigarette smoke-induced chronic bronchitis, naringin inhibited the protein and gene expression of IL-6, IL-8, IL-1β, IL-8, TNF, and leukotriene B4 (LTB4) (Luo et al., 2012). Naringin also inhibits TNF-α induced NADPH oxidase-4/NF-κB signaling pathway in human umbilical vein endothelial cells and activates the PI3K/AKT signaling pathway, thereby suppressing TNF-α induced oxidative stress and inflammatory responses (Li et al., 2014). Cheng et al. (2017) found that naringin improved the survival rate of random skin flaps in rats; the naringin group had significantly lower levels of TNF-α and IL-6 and a significantly higher level of SOD group than the control group, indicating that naringin can reduce inflammatory responses and oxidative stress (Cheng et al., 2017). Naringin could also reduce ischemia-reperfusion injury in extremity trauma combined with acute vascular complications and prolonged the use of a tourniquet in extremity surgery (Gürsul et al., 2016). In some chronic inflammatory disease experiments, naringin was found to have anti-inflammatory and antioxidant effects in a rat air sac inflammation model (Jain and Parmar, 2011). Studies have demonstrated a significant inhibitory effect of naringin on interchondral joint damage and inflammatory cell infiltration (Kawaguchi et al., 2011). Another study revealed the protective effects of naringin against dextran sodium sulfate-induced ulcerative colitis in mice (Cao et al., 2018).

In summary, naringin can improve inflammation in experimental animals, and its associated mechanism is now relatively clear. Non-etheless, there are only a few animal experimental models to study the ischemia-reperfusion of skin flaps, and its specific mechanism needs to be studied further. According to the study, naringin may exert anti-inflammatory effects by regulating the expression of inflammatory factors such as IL-6 and TNF-α, which can mitigate both the inflammatory response and the ischemia-reperfusion injury of the flap. Concurrently, naringin may play the antioxidative role in the flap ischemia-reperfusion process by regulating various antioxidant enzymes and may inhibit the inflammatory response by scavenging free radicals and increasing SOD *in vivo*. In conclusion, naringin has obvious advantages in inhibiting the inflammatory response to flap ischemia-reperfusion to thus improve flap survival. Further experimental studies in this direction should be conducted in the future.

## 4 Naringin accelerates wound healing after flap repair and improves the ability of the flap to resist infection

Wound healing occurs in four stages, namely, hemostasis, inflammation, proliferation, and remodeling; these are overlapping and interrelated processes (Diegelmann and Elvans, 2004). Disruption of any mechanism, such as circulatory factors, inflammatory irritation, local ischemia, and hypoxia during wound healing, leads to delayed wound healing. In recent years, the expression of pro-inflammatory factors such as TNF-α, IL-1β, and IL-6 has been found to be highly correlated with wound healing (Wang et al., 2022). In the acute and early stages of trauma, pro-inflammatory factors can exert a pro-inflammatory effect, enhance the immune response, stimulate epithelial growth around the trauma, and exert a myogenic effect. However, too high concentrations of pro-inflammatory cytokines or too low concentrations of anti-inflammatory cytokines lead to abnormal inflammation of the wound, inhibiting the healing process and leading to the formation of chronic hard-to-heal wounds. In the case of an unreasonable flap design with too large aspect ratio, the distal osmotic pressure is insufficient, the reflux around the flap is poor, and the distal end is prone to necrosis; this can easily result in necrosis of the part of the flap after repair and lead to the non-healing of the wound. The problem is usually solved clinically by using wound dressing, skin grafting, reuse of the original flap, and designing another flap repair, but each of these methods has its own limitations. Naringin is found to be of significant advantage in wound healing. In fact, naringin-loaded gum Arabic and pectin hydrogels promoted wound angiogenesis, re-epithelialization, and collagen deposition to accelerate wound healing, significantly down-regulated the expression of inflammatory mediators TNF- α and apoptosis, and exhibited antioxidant properties, demonstrating the therapeutic effect of naringin on wound repair (Alsakhawy et al., 2022). Naringin ointment preparations were found to have wound healing potential by down-regulating the expression of inflammatory factors, including NF-κB, TNF-α, IL, apoptotic factors pol-γ and Bcl2 associated X, and upregulating the expression of growth factors VEGF and TGF-β, thereby regulating collagen -1 expression, inducing angiogenesis, and promoting wound healing (Kandhare et al., 2016). The exact mechanism by which naringin treatment improves wound healing may be explained in part by its combined ability to enhance cell migration, activate the VEGF pathway, and upregulate the downstream effector MMP-2 and MMP-9 (Yen et al., 2022). In depilated mice, naringin hydrogel had a therapeutic effect on fully covered wounds of dorsal resection rectangular full-thickness flaps, demonstrating that it inhibits NF-kB and cyclooxygenase-2 activation and may promote deep dermal wound repair and regeneration (Vabeiryureilai et al., 2022). In another study, naringin could inhibit the proliferation and migration of fibroblasts while promoting the apoptosis of fibroblasts, effectively inhibiting the growth and motility of hypertrophic scar fibroblasts by inhibiting the phosphorylation of Akt, and could thus be a potential new drug for the treatment of hypertrophic scar (Song et al., 2018). This study demonstrated the obvious advantages of naringin in repairing wounds, effectively solving the problem of partial non-healing of wounds after flap repair and providing a new strategy for the future clinical treatment of non-healing wounds post-flap repair.

Incomplete debridement of emergency wounds or ineffective control of infected wounds aggravates the occurrence of infection and may result in the non-healing of wounds or even affect flap survival after flap repair. Naringin was found to have antimicrobial effects against periodontal pathogens *in vitro*, and at low concentrations, it also inhibited common oral microorganisms (Tsui et al., 2008). In another study, naringenin enhanced the effects of ciprofloxacin and tetracycline on the *Pseudomonas aeruginosa* biofilm (Dey et al., 2020).

Thus, naringin has the potential to promote wound healing, and its mechanism of regulation may be related to the NF-κB signaling pathway, which inhibits inflammatory factors such as TNF-α and IL-6 to thus promote wound healing, which has important utility for the clinical treatment of partial wound non-healing after flap repair. However, the specific mechanism still needs to be studied in the future. Naringin can be used alone as an antibacterial or in combination to improve the efficacy of other antibacterial drugs, which is of reference value for future antibacterial treatment in the clinic.

## 5 Enhancement of naringin bioavailability by nanoformulation

Studies have identified a variety of biological activities of naringin; however, the poor solubility of naringin in an aqueous solution severely limits its bioavailability (Hsiu et al., 2002; Tang and Yan, 2016). The problem of low bioavailability of naringin has been solved by designing a variety of different nanocarriers to improve the solubility, stability, and controlled release time of naringin bioactive substances to achieve the bioactivity of naringin by relatively small amounts and to avoid the waste of biological resources and side effects induced by high doses. Metal nanoparticles are advantageous for drug delivery, therapeutic and diagnostic applications, and their controlled size and structural surface composition make them versatile nanocarrier systems to enhance the therapeutic efficacy of drug delivery. Gallotannin-capped gold nanoparticles could increase the concentration of naringin, which ultimately enhanced the bactericidal activity against Gram-positive and Gram-negative bacterial strains, confirming that naringin exerts its bactericidal activity through cell membrane disruption and cellular breakdown of bacteria (Rao et al., 2017). Ternary nanoparticle-naringin inclusion complex can effectively encapsulate and deliver naringin, and *in vitro* release assays show that these complexes can improve the delayed release of naringin in the simulated intestinal mucosa (Feng et al., 2017). In another study, encapsulation of naringin in poly (lactic acid)-hydroxyacetic acid copolymer using the emulsification-diffusion-evaporation method improved the drug formation and stability, and enhanced the bioavailability of naringin (Ghosal et al., 2018). Methoxy poly (ethylene glycol)-poly (ε-caprolactone) (MPEG-b-PCL) is an amphiphilic biodegradable and biocompatible diblock copolymer that acts as a nanomicrosphere carrier and enhances solubility. MPEG-PCL micelles containing naringin can be used as an effective drug delivery system with significantly improved solubility of naringenin and faster and better drug release properties. The safety of MPEG-PCL *in vivo* was confirmed in cytotoxicity and histopathological assays in oral cells and mucosa (Fan et al., 2020). Another study showed that a high concentration of ovalbumin (OVA)-carboxymethyl konjac glucomannan (CKGM) nano-delivery system enhances low water-soluble bioactives and improves the bioavailability of naringin (Tang et al., 2022).

In summary, poor water solubility and low bioavailability of naringin are the main concerns for its use as a therapeutic agent. Nanodelivery systems have the ability to break through the pharmacokinetic limitations of naringin and improve the pharmacokinetic and pharmacological properties of naringin to maximize its therapeutic potential by using nanoparticles, micelles, and nanoemulsion formulation drug delivery systems. Until now, no study has been reported on naringin nanopreparations to improve flap survival, and the development and application of naringin nanopreparations need to be further explored in the future.

### 5.1 Conclusions and future perspectives

Partial or complete necrosis of the flap remains a clinical challenge. Earlier studies have identified various drugs and biological factors, such as bivalirudin (Cai et al., 2017), bezafibrate (Lin et al., 2016), sildenafil (Sarifakioglu et al., 2004), VEGF (Kryger et al., 2000), etc., that increase vascular permeability, promote angiogenesis, reduce ischemia-reperfusion injury, and prevent thrombosis, thus improving flap viability. In recent years, through in-depth research and development, domestic and foreign researchers have discovered the use of phytochemicals that can treat flap necrosis. In fact, it is gradually becoming a hot spot for research and may become the future development direction for the treatment of flap necrosis. Current studies have shown that naringin can stimulate angiogenesis and related cell proliferation and apoptosis by regulating the NF-κB and PI3K/AKT signaling pathways, inhibiting the ischemia-reperfusion inflammatory response and oxidative stress, providing a growth environment for vascular regeneration and flap healing, and effectively promote vascular regeneration and accelerate wound healing after flap repair. The potential use of naringin for the clinical treatment of flap necrosis will reduce the pain of patients who have to undergo secondary surgery after flap necrosis, reduce their financial burden, and significantly impact the future treatment of flap necrosis. Although research on the pharmacological effects of naringin has yielded some results, the specific mechanism of its pharmacological effect on improving the survival rate of skin flaps is not yet clear, or only a few of the related signaling pathways have been identified. Besides stimulating vascular growth factors to promote neovascularization, in what other ways does naringin stimulates new vessel growth to improve flap survival? What other mechanisms does naringin use to reduce inflammation and promote flap survival during flap ischemia-reperfusion? In the case of partial flap necrosis after flap repair and chronic wound non-healing, what is the best way to achieve optimal wound healing by topical application: intravenous injection or a combination of both? Does naringenin reduce the rate of postoperative infection in skin flap grafts, and what are the mechanisms involved? These questions need to be further investigated in future experiments.

Studies have shown that naringin given orally to rats improves the survival of randomized skin flaps (Cheng et al., 2017), but poor oral absorption of naringin, short half-life, unstable blood concentrations, and poor bioavailability severely hinder its clinical application (Chen et al., 2019). If the pharmacological effects and clinical indications of naringin to improve flap survival remain unchanged as a premise, the modification of naringin-based agents may have significant implications for its clinical application in the treatment of flap necrosis. The nano-delivery system can overcome the pharmacokinetic limitations of naringin, improve its bioavailability and enhance its solubility, which is important for its future clinical application in the treatment of skin flap necrosis. However, many questions about nanotechnology still need to be solved, such as the selectivity, stability, toxicity, and degradability of nanomaterials, and the application of nanotechnology to naringin to improve the survival rate of skin flaps needs to be further explored. Current studies on naringin only focus only on absorption, *in vivo* processes, and drug interactions in rats, and there is a lack of research on large and medium-sized animals that are physiologically close to humans, and there is a need to strengthen research in this area.

In conclusion, naringin has the therapeutic potential to improve flap survival by promoting new blood vessel regeneration, inhibiting ischemia-reperfusion inflammatory response of the flap, and accelerating healing after flap repair. These activities of naringin provide new ideas and directions for future clinical treatment and prevention of flap necrosis and has a key clinical application value, but the detailed mechanism involved needs to be studied more exhaustively.

## Abbreviation

VEGF, Vascular endothelial growth factor; SOD, Superoxide dismutase; MDA, Malondialdehyde; IL-6, Interleukin-6; IL-8, Interleukin-8; IL-1β, Interleukin-1; LTB4, leukotriene B4; TNF-α, tumor necrosis factor-α; HUVECs, Human umbilical vein endothelial cells; DSS, Dextran Sulfate Sodium Salt; PLGA, poly(lactic-co-glycolic acid).
